# Postzygotic Isolation Evolves before Prezygotic Isolation between Fresh and Saltwater Populations of the Rainwater Killifish, *Lucania parva*

**DOI:** 10.1155/2012/523967

**Published:** 2012-01-30

**Authors:** Genevieve M. Kozak, Arthur B. Rudolph, Beatrice L. Colon, Rebecca C. Fuller

**Affiliations:** ^1^Department of Animal Biology, University of Illinois, Champaign, IL 61820, USA; ^2^Department of Biology, University of Florida, Gainesville, FL 32611, USA

## Abstract

Divergent natural selection has the potential to drive the evolution of reproductive isolation. The euryhaline killifish *Lucania parva* has stable populations in both fresh water and salt water. *Lucania parva *and its sister species, the freshwater *L. goodei*, are isolated by both prezygotic and postzygotic barriers. To further test whether adaptation to salinity has led to the evolution of these isolating barriers, we tested for incipient reproductive isolation within *L. parva* by crossing freshwater and saltwater populations. We found no evidence for prezygotic isolation, but reduced hybrid survival indicated that postzygotic isolation existed between *L. parva* populations. Therefore, postzygotic isolation evolved before prezygotic isolation in these ecologically divergent populations. Previous work on these species raised eggs with methylene blue, which acts as a fungicide. We found this fungicide distorts the pattern of postzygotic isolation by increasing fresh water survival in *L. parva*, masking species/population differences, and underestimating hybrid inviability.

## 1. Introduction

There is substantial evidence that adaptation to different environments can lead to the evolution of reproductive isolation between populations, a process referred to as ecological speciation [1–6]. Ecological speciation predicts the evolution of both prezygotic and environmentally dependent postzygotic isolation. Prezygotic isolation can evolve as mating signals and preferences adapt to different environments [7–12]. Extrinsic (environmentally dependent) postzygotic isolation may also evolve since hybrids have intermediate phenotypes and are poorly adapted to parental habitats [13–17]. Currently, there is less evidence that genetic incompatibilities between populations (intrinsic postzygotic isolation) can evolve simply as a consequence of adaptation to different habitats [18–20]. Most identified intrinsic isolating barriers have no clear relationship to adaptation and may have arisen subsequent to ecological divergence [21–23]. However, theoretical and empirical work suggests intrinsic isolation can arise through ecological divergence if there are epistatic interactions between alleles conferring environment-specific adaptations [24–26].

 When prezygotic, extrinsic, and intrinsic postzygotic reproductive isolating barriers evolve as byproducts of adaptation, the probability that they will lead to speciation depends on their cumulative strength and ability to persist in the face of gene flow when incipient species come into contact [27]. If the cumulative strength of isolating barriers is insufficient, population divergence will be lost via introgression, and speciation will not occur [28]. Therefore, determining how adaptation generates both pre- and postzygotic isolating barriers and how rapidly these barriers evolve is a key focus of speciation research.

 Much previous work has focused on timing and order in which reproductive isolating barriers arise, but has given little consideration to their ecological context. In species of *Drosophila* studied in a common laboratory environment, prezygotic isolation evolves faster than postzygotic isolation [29, 30]. However, this effect seems to be driven by the effect of sympatry and, in allopatric species, pre- and postzygotic isolation evolve at the same rate. Prezygotic isolation also evolves well before postzygotic isolation in birds [31, 32], salamanders [23], and several groups of fish (including centrarchids [33], African Rift Lake cichlids [34], and darters [35, 36]). For instance, postzygotic isolation in fish appears to accumulate slowly with hybrid inviability not becoming complete until species have been separated for 10 to 20 million years [33, 34]. However, in many of these studies, hybrids are raised in a common laboratory environment, which may underestimate hybrid inviability. Differences in population ecology and how these may relate to the strength of isolating barriers are not usually considered. One exception to this is work on stickleback fish which has found that young stickleback species pairs exhibit prezygotic and environmentally based postzygotic isolation, while older pairs show both prezygotic and intrinsic postzygotic isolation [8, 13, 37, 38].

 In our study, we ask which reproductive isolating barriers have evolved between ecologically divergent populations within one species of killifish and compare them to barriers that have evolved between two sister species. This allows us to determine the order in which isolating barriers arise as populations adapt to different ecological conditions. The rainwater killifish, *Lucania parva*, is a euryhaline species with permanent populations existing in fresh, brackish, and salt water across the Southeastern United States [39]. *L. parva*'s sister species, the bluefin killifish (*Lucania goodei*), is found almost exclusively in fresh water in Florida [40]. Sympatric populations of *L. parva* and *L. goodei* can be found in several freshwater sites across Florida. Multiple lines of evidence suggest that adaptation to different salinity conditions has occurred between species. *L. goodei *has higher fitness in fresh water relative to *L. parva*, and *L. parva *fares better in brackish and salt water than *L. goodei *[41]. Additionally, *L. goodei* has a decreased rate of hatching success at high salinities while *L. parva* has a lower rate of survival to adulthood in fresh water [41–43]. However, *L. parva* appears to have equal hatching success with *L. goodei* in fresh water. All this previous work on *L. parva* and *L. goodei* has raised eggs with the fungicide methylene blue [44]. While this fungicide improves hatching success, it may do so disproportionally for different salinities, populations, or species. Therefore, in our study, we raised eggs in water with and without methylene blue.

 Reproductive isolation between *L. parva* and *L. goodei* involves both prezygotic and postzygotic barriers. Behavioral isolation is quite strong with *L. parva* and *L. goodei* mating pairs taking longer to produce eggs than conspecific pairs and producing fewer eggs [41]. Postzygotic isolation between species is both extrinsic and intrinsic. Backcrosses, F1, and F2 hybrids have reduced survival, particularly at high salinities. In addition, F1 hybrids sons of *L. parva* females and *L. goodei* males have reduced fertility [43].

 Some of these isolating barriers between *L. parva* and *L. goodei* may have arisen due simply to adaptation to fresh and salt water. Life in fresh water and salt water pose different osmoregulatory challenges for aquatic animals. In fresh water, fish need to keep excess water out of their bodies, while retaining vital salts. However, marine fish need to extricate salt, but retain water [45]. Throughout the life of the fish, osmoregulation can occur in the gills, guts, kidneys, and skin [46]. Therefore, adaptation to salinity can potentially cause divergence in many genes involved in ion regulation [47, 48], increasing the likelihood of speciation as a direct consequence of adaptation to salinity [5]. To ask how salinity may drive the evolution of isolation barriers in *Lucania*, we measured isolation between *L. parva *populations adapted to different salinity environments.

 We collected *L. parva* from a permanent fresh water population (Pecos River) and a salt water population (Indian River Lagoon). We crossed Pecos and Indian River fish and predicted that if prezygotic isolation existed, between populations, mating pairs would take longer to mate and produce fewer eggs than within population pairs. We then raised Pecos-Indian River hybrid eggs in five water chemistries ranging from fresh to salt water and measured survival. If postzygotic isolation exists, hybrid eggs and fry should have lower survival than either of the parental populations. Hybrid inviability across environments would be evidence for intrinsic isolation, while environmentally dependent inviability would suggest that isolation is extrinsic. Furthermore, if any local adaptation is present, we would predict the freshwater population to have higher survival than the saltwater population in fresh water treatments and the saltwater population to have higher survival in salt water conditions. We measured survival of our *L. parva* populations with and without methylene blue to determine if the fungicide had any effect on measures of postzygotic isolation.

 Additionally, we wished to compare the survival of freshwater, saltwater, and hybrid *L. parva* to *L. goodei* survival. Previous work has established that *L. goodei* has extremely low survival in salt water, but equal survival with *L. parva* in fresh water. However, in these studies, eggs were raised in methylene blue, and only a single fresh water treatment was used [39]. Therefore, we collected eggs from one population of *L. goodei *and raised them in two fresh water treatments in the absence of methylene blue. We predicted that *L. goodei* should have higher survival in fresh water than *L. parva*.

## 2. Methods

We collected *L. parva* from two ecologically different and geographically distant sites: an inland river in Texas and the Atlantic Ocean off the coast of Florida. Our freshwater site was Pecos River, along the Pecos-Crockett County border, TX. At the time of collection, the carbonate hardness (KH, a measure of mineral content) of the water was low (between 3 and 4). However, the upper Pecos River does have a history of salinization due to input from salt springs and dam construction altering water flow, which may contribute to *L. parva*'s persistence there [49]. Our saltwater site was Indian River Lagoon, Brevard County, FL, on the Atlantic coast. Salinity in Indian River is typically 35 ppt. We collected *L. goodei *from the Wakulla River, Wakulla County, FL. At each site, we collected animals using dipnets and seines. The collected fish were transported back to University of Illinois and housed in 75–109 L stock tanks. Indian River (IR) fish were kept in reverse osmosis water raised to 35 ppt salinity using Instant Ocean Sea Salt (Spectrum Brands, Atlanta, GA). They were then transitioned to 10 ppt water, then at the beginning of the experiment to tanks containing city water treated with the dechlorinating agent Start Right (Jungle Laboratories, Cibolo, TX) at 2 ppt salinity. Pecos River fish were kept in treated city water at 2 ppt salinity and with Alkaline Regulator (Seachem, Madison, GA) added to bring the carbonate hardness (KH) to 10. Fish were fed daily *ad libitum* with a mixture of frozen brine shrimp and flake food. Fish were maintained under a light cycle of 14 hours light, 10 hours dark.

We performed both within and between population crosses. We set up four different cross types: two within population crosses (Pecos female by Pecos male, IR female by IR male) and two between population crosses (Pecos female by IR male, IR female by Pecos male). There were 8 replicates of each cross type, for a total of 32 pairings. For each pair, we placed one male and one female in a 38 L tank filled with dechlorinated city water at 2 ppt salinity. Visual barriers were placed between all tanks to isolate mating pairs from others. Four yarn mops were provided as a spawning substrate (two floating and two sinking mops).

 The mops were checked for eggs every 2-3 days. All collected eggs were checked under a microscope to verify that they were recently fertilized. Killifish eggs take approximately 7–9 days to hatch; therefore, most eggs (at 1-2 days old) were very early in development when they were transferred to their water treatments. We recorded the number of eggs found on each egg check. Latency to mate was measured over the first 47 days and was calculated as the number of days until the first egg was found. If a pair had not mated after 47 days, we assigned them a latency of 48 days (the total number of days plus 1 day) [41]. After 47 days, we removed visual barriers between tanks to encourage spawning and continued collecting eggs. We summed the total number of eggs laid over the entire experiment (61 days).

 Eggs were transferred to small plastic tubs with different water treatments. There were three fresh water treatments: pure reverse osmosis water (RO), soft water (KH3), and hard water (KH8). The RO water was created using a filtration system that removes sediment, chlorine, and other large ions from city water (AquaFx Barracuda 4 Stage RO/DI System, Winter Park, FL). Soft water was created by adding Alkaline Regulator (Seachem, Madison, GA) and R/O Right (Kent Marine, Franklin, WI) to adjust the ionic content of RO water to a carbonate hardness of KH3. Hard water was created by adding Alkaline Regulator and R/O Right to dechlorinated city water until its hardness was KH8. The salt water treatments were made by adding Instant Ocean Sea Salt to RO water until the desired salinity was reached. Ocean water is typically 32 ppt, and we used two salinity treatments: saline (20 ppt) and hypersaline (40 ppt). Additionally, we raised some eggs in the KH8 and 20 ppt treatments with methylene blue, the antifungal agent. A 3 ppm solution of methylene blue (C_16_H_18_N_3_SCl; Kordon LLC, Hayward, CA) was added to the water immediately after eggs were placed in it. We rotated the water treatment every egg collection day to assure an equal distribution of eggs in each water treatment. We collected eggs until each water treatment had at least 10 eggs from each tank. Once the eggs hatched, we transferred the fry into clean tubs with the same water treatments.

 Eggs and fry were censused every 2-3 days. We recorded the number of eggs that were alive or dead, the number of eggs hatched, and the number of fry that were alive or dead. These censuses continued until 14 days after hatching, at which point fry were euthanized with an overdose of MS-222. We measured survival in several ways. We measured the proportion of eggs that hatched (hatching success), the proportion of fry that survived to 14 days of age (fry survival), and the proportion of eggs that produced surviving fry of 14 days of age (total survival). These proportions were calculated separately for each family in each water chemistry. We combined the data for the two between population cross types into one hybrid group. We had eggs from 32 families total (Pecos = 8, IR = 8, Hybrid = 16). Not all families had eggs in all water chemistries; therefore, we list sample sizes for each water chemistry in our table legends.

 To measure *L. goodei* survival in fresh water in the absence of methylene blue, we collected eggs from *L. goodei* stock tanks (not from the preestablished crosses). Mops from these tanks were checked three times a week. The eggs collected were placed into KH3 or KH8 treatments, both without methylene blue. These eggs were also checked under a microscope to verify they were fertilized. Hatching success and fry survival were measured as described above.

 All statistical analyses were performed using SAS statistical software (SAS V 9.1, Cary, NC). Measures of prezygotic isolation (latency to mate and total number of eggs produced) were analyzed in a general linear model with male source population (Pecos, Indian River), female source population (Pecos, Indian River), and the interaction between male population and female population. If behavioral isolation existed, we would expect a significant interaction between male and female population. There was an outlier in our latency to mate data, with one Indian River female by Pecos male taking more than 47 days to mate, so we performed the analysis with and without this outlier to determine if it affected our conclusions.

 For survival data, we analyzed the proportion surviving at each life stage for each cross type using generalized linear models assuming a binomial distribution (proc genmod in SAS) and used maximum likelihood to evaluate the significance of effects. We used the “dscale” option in SAS to control for overdispersion when this occurred [43]. To determine survival in the absence of methylene blue, we used a model that considered the effects of water chemistry (RO, KH3, KH8, 20 ppt, 40 ppt), cross type (Pecos, IR, Hybrid), and their interaction on the probability of hatching, fry survival, and total survival. We also ran analyses where we included family (nested within cross type) as a repeated factor in our general linear model, but it did not alter our results, and these analyses are not presented here.

 To determine the effects of methylene blue on survival in hard and 20 ppt, we ran a second model which examined the effects of cross type, water chemistry (KH8, 20 ppt), presence/absence of methylene blue and their interactions on the probability of hatching, fry survival, and total survival.

 To compare *L. parva* to* L. goodei* fresh water survival in the absence of methylene blue, we analyzed probability of survival at each stage (egg, fry, total) in fresh water chemistries (KH3, KH8), based on cross type (Pecos, IR, Hybrid, *L. goodei*) and included the interaction between cross type and water chemistry. Means and standard errors are reported throughout for all analyses.

## 3. Results

**Figure 1:**
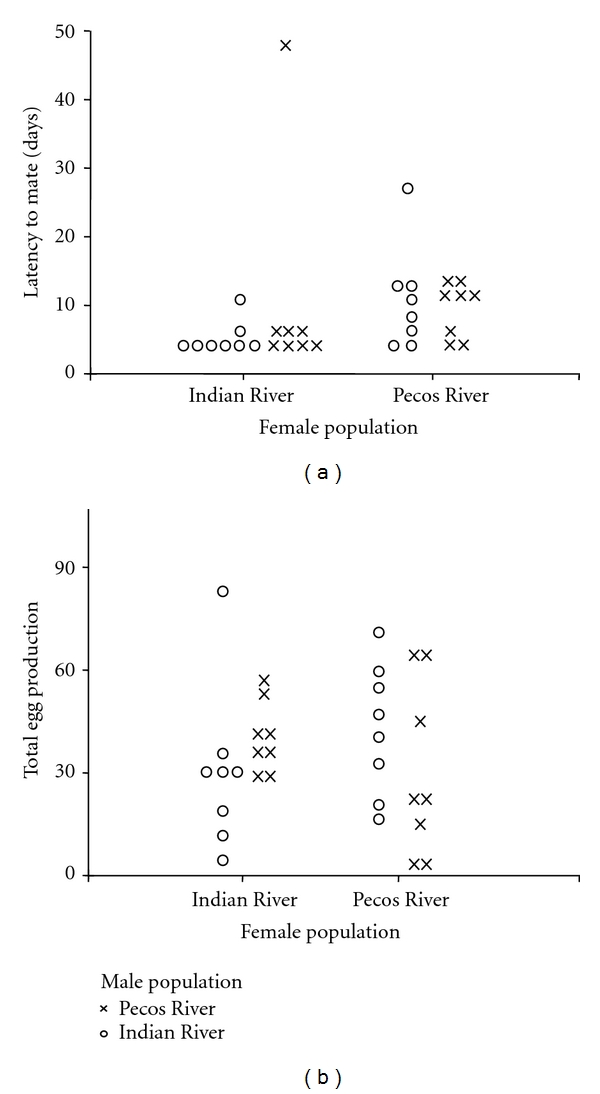
*L. parva *mate preference. Measures of behavioral isolation between populations plotted by female source population. Circles indicate females mated to Indian River males, crosses indicate females mated to Pecos River males. (a) Latency to mate in days (including outlier), (b) total egg production over 61 days.

We found no evidence for prezygotic isolation between Pecos and Indian River fish. Between populations pairs (Pecos male by Indian River female; Indian River male by Pecos female) did not differ from within population pairs in latency to mate or total number of eggs produced (Table 1; Figure 1). Removal of the latency outlier did not alter our conclusions about prezygotic isolation. However, when the outlier was removed, we found there was a difference in latency to mate between female populations with Indian River females mating sooner than Pecos females (IR = 5.00 + 1.89 days, Pecos = 9.94 + 5.82 days).

**Figure 2:**
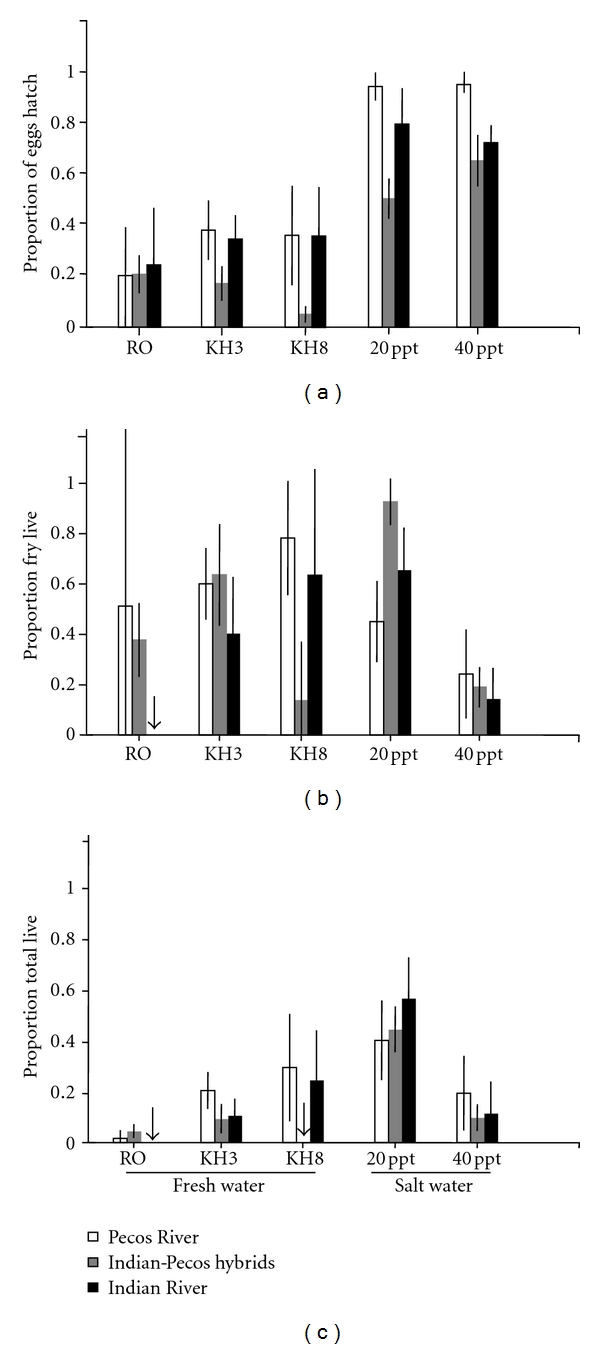
*L. parva* survival differences in fresh and salt water. Mean survival probabilities (+ standard error) for Pecos River (white bars), Indian River (black bars), and Indian-Pecos hybrid crosses (gray bars) across different water chemistries: reverse osmosis water (RO), soft water (KH3), hard water (KH8), saline (20 ppt), and hypersaline (40 ppt). All crosses were raised in the absence of methylene blue. Arrows indicate mean survival probability of zero. (a) The proportion of eggs hatched, (b) proportion of fry that survived to 14 days after hatch, (c) total survival (proportion of eggs that survived to 14 days after hatch).

**Figure 3:**
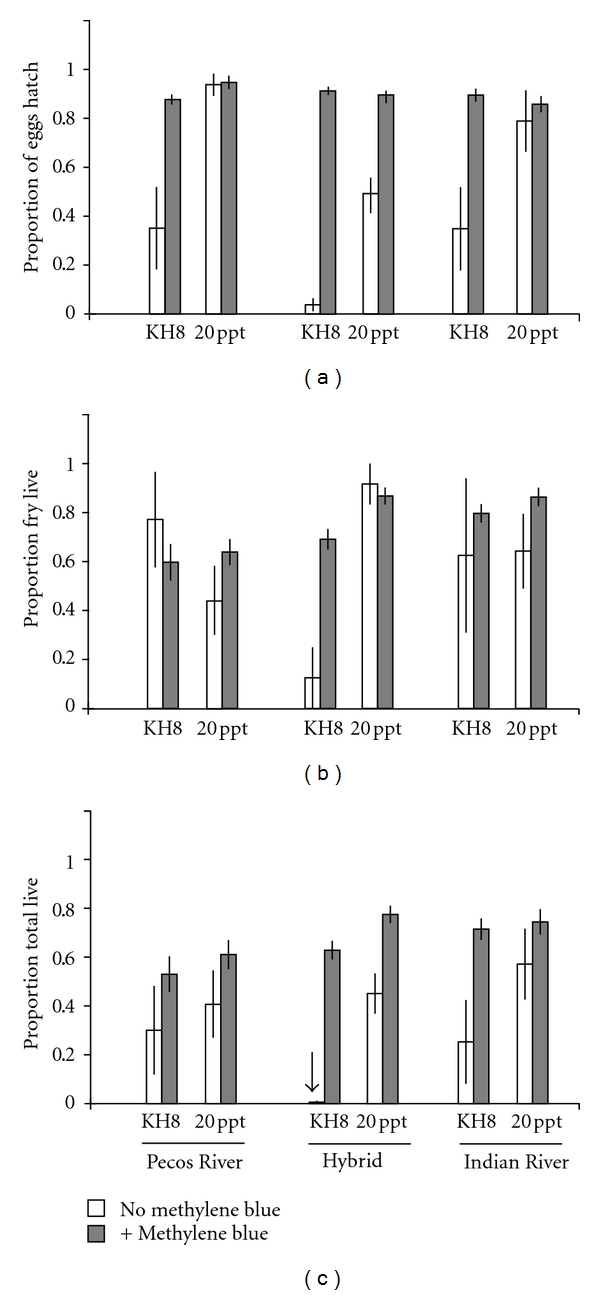
*L. parva* survival differences with methylene blue addition. Mean survival probabilities (+ standard error) for Pecos River, Indian River, and Indian-Pecos hybrid crosses in fresh water (KH8) and salt water (20 ppt) with methylene blue addition (gray bars) and without (white bars). Arrows indicate mean survival probability of zero. (a) The proportion of eggs hatched, (b) proportion of fry that survived to 14 days after hatch, (c) total survival (proportion of eggs that survived to 14 days after hatch).

Despite a lack of prezygotic isolation, we found that offspring from Pecos-Indian River hybrid crosses had reduced survival. Hybrid eggs had lower hatching success than within population eggs across different water treatments (Table 2(a), Figure 2(a)). The proportion of fry that lived and total survival were also lower for hybrid crosses, but only in hard water (Tables  2(b) and 2(c); Figures 2(b) and 2(c); significant cross by water treatment interaction). However, no reduction in hybrid hatching rates was detected when methylene blue was added to the water treatments (Table 3, Figure 3; significant methylene blue by cross interaction). When methylene blue was present, hybrid offspring survived quite well. There was little evidence for local adaptation in egg and fry survival as we did not detect consistent differences between Pecos and Indian River survival. In both populations, hatching success was higher in salt water than in fresh water. 

**Figure 4:**
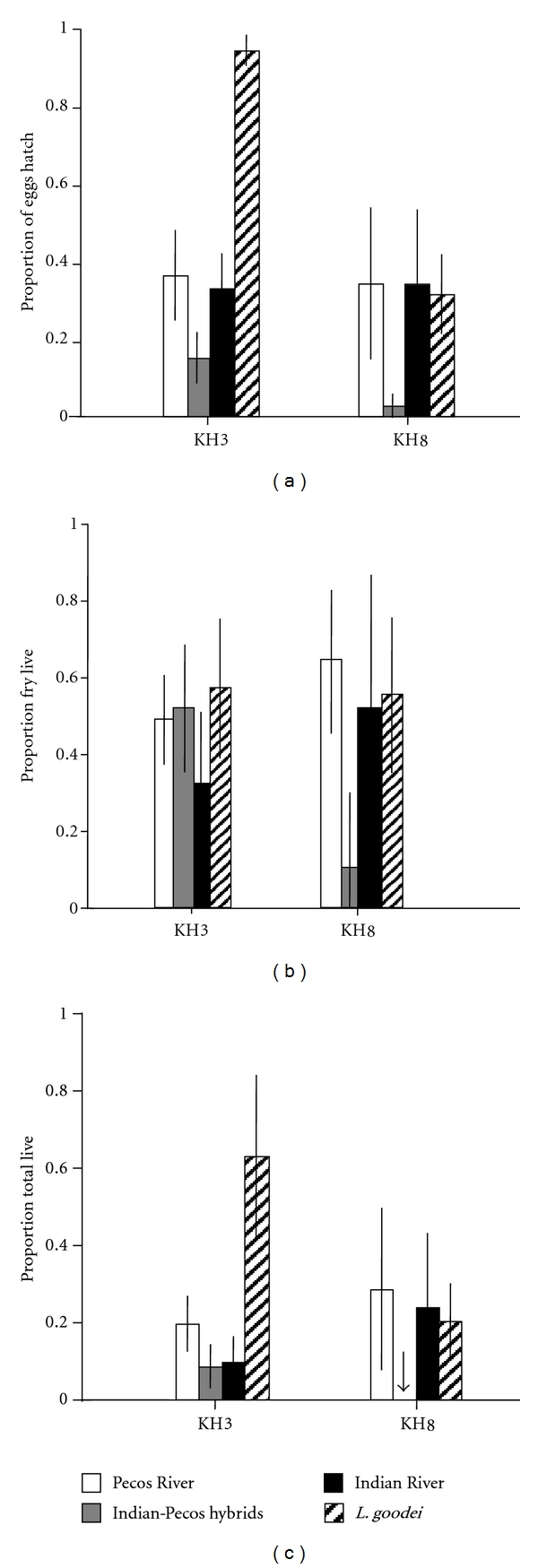
*L. parva* and *L. goodei* survival differences in fresh water. Mean survival probabilities (+ standard error) for Pecos River (white bars), Indian River (black bars), Indian-Pecos hybrids (gray bars), and *L. goodei* (hatched bars) crosses in soft (KH3) and hard fresh water (KH8). All crosses were raised in the absence of methylene blue. Arrows indicate mean survival probability of zero. (a) The proportion of eggs hatched, (b) proportion of fry that survived to 14 days after hatch, (c) total survival (proportion of eggs that survived to 14 days after hatch).

*L. goodei* eggs hatched more than *L. parva* eggs in fresh water treatments in the absence of methylene blue (Table 4; Figure 4). Total survival of *L. goodei* eggs and fry was also higher than *L. parva* survival in fresh water. These differences seem primarily driven by high survival of *L. goodei* eggs and fry in soft water treatments. These results are in contrast to previous work that found no difference between the species when methylene blue was used.

## 4. Discussion

Here, we show that postzygotic isolation has begun to evolve between freshwater and saltwater populations of *L. parva*. However, there is no evidence that any prezygotic isolation yet exists. This suggests that genes involved in hatching success and fry survival evolve more rapidly between *L. parva* populations than genes involved in mating traits and preferences. Most previous work suggests that pre- and postzygotic isolation evolve at similar rates in allopatric populations [29, 30], but this does not appear to be true in *L. parva*.

When we examined population differences within *L. parva*, we found that F1 hybrids between freshwater and saltwater populations had reduced survival compared to offspring from within population crosses. These effects were most apparent in challenging water chemistries: in fresh water and in the absence of methylene blue. The most drastic reduction of hybrid survival was in hard water (KH8). The lethality of hard water may be due to fungus that grew readily in this water treatment. Fungal infections are a major source of egg mortality and both high salinity and methylene blue can prevent infection, although methylene blue is more effective [44, 50]. Methylene blue may also add ions to the water, which may decrease osmoregulatory stress and may be why methylene blue also increased fry survival at low salinities. This suggests that hybrid eggs were less viable than eggs from within population crosses and physiologically challenging water chemistries revealed this decreased viability. We also showed that *L. parva* has lower survival compared to *L. goodei* in fresh water in the absence of methylene blue. This contradicts previously published results that used methylene blue and found no difference in fresh water survival between species [41–43]. This suggests that F1 hybrids between *L. goodei* and *L. parva* may also have low survival in fresh water, but these effects have been masked by the use of methylene blue in previous studies and hybrid fitness may have been previously overestimated.

 The decreased viability of Pecos and Indian River hybrid offspring suggests that intrinsic postzygotic isolation exists between populations. The main difference between Pecos and Indian River populations is their native salinity, suggesting that genetic incompatibilities have arisen as a byproduct of adaptation to saline environments. However, in allopatric populations, any mechanism which causes unique alleles to become fixed has the potential to cause incompatibilities as novel alleles come into contact and interact in hybrids (Dobzhansky-Muller incompatibilities: [18, 51–53]). Intrinsic postzygotic isolation between populations can also arise due to genetic drift [20, 52, 54] or genomic conflict [55]. Pecos and Indian River are geographically distant, separated by more than 2400 km and the Gulf of Mexico. Work on other related species from the *Fundulus* clade has found substantial divergence between east and west Gulf populations, possibly due to genetic drift [56]. Therefore, we are currently working on determining the degree of genetic divergence and phylogenetic relationship between these populations using sequence data. In addition, we are conducting crosses between other ecologically divergent populations from the same geographical region as well as geographically distant but ecologically similar populations. This ongoing work will determine if hybrid inviablity in *L. parva* evolves primarily due to salinity adaptation rather than due to drift.

 How isolation arises during the initial stages of speciation, when a single population splits into two and populations begin to diverge, still represents a “missing link” in speciation research [57]. By showing that intrinsic postzygotic isolation has begun to evolve between divergent *L. parva* populations, our work suggests it may have been the first barrier to arise between *L. parva* and its sister species *L. goodei*. Adaption to salinity is primarily physiological and, therefore, may be particularly likely to cause intrinsic isolation through epistatic interactions. Similarly, physiological changes associated with toxic environments also appear to lead to substantial genetic changes between populations and, in some cases, to hybrid inviability [58–61]. Therefore, physiological adaptation may be a primary force leading to postzygotic incompatibilities.

 Currently, there are competing ideas about how isolating barriers evolve during speciation. In one proposed scenario, strong prezygotic isolation evolves before strong postzygotic isolation. Thus, prezygotic isolation plays a primary role in preventing interbreeding, and postzygotic isolation slowly completes the process of speciation as decreased hybrid fitness and irreversible genetic incompatibilities accumulate [28, 62, 63]. However, this conclusion is based on studies of species pairs that have already undergone speciation [29, 34, 35], populations that occur in sympatry where reinforcement may have strengthened prezygotic isolation [30, 64, 65], or populations in which feeding and mating occur in the same habitat (such as phytophagous insects [66–68]). Nevertheless, some incipient species do show prezygotic isolation without any postzygotic barriers [57, 69, 70].

 There is another possible route to speciation. Genetic divergence might produce hybrid inviability between populations and prezygotic isolation evolves subsequently as divergence continues or as incipient species come into sympatry and reinforcement occurs [18]. When natural selection drives genetic divergence between populations, evolving postzygotic isolation should be primarily environmentally dependent. Many examples of adaptation to divergent environments producing extrinsic isolation exist [1, 4, 15, 17, 19, 71–73], while there are few examples for intrinsic isolation. In a survey of 20 ecologically divergent species pairs, all species exhibited some prezygotic isolation and extrinsic postzygotic isolation, but only three pairs had any documented intrinsic postzygotic isolation [57]. Intrinsic isolation as a result of ecological divergence has only been substantially documented in dwarf and normal lake whitefish [74, 75], copper tolerant plants [58], and an experimental evolution study in yeast [26]. However, few studies distinguish between extrinsic inviability and intrinsic inviability that appears under stressful conditions [62], such as the decreased viability that appeared in challenging water chemistries in our study. Therefore, future work needs to establish the contribution to divergence of both extrinsic and intrinsic postzygotic isolation and the underlying genetic basis of both. Such work will allow us to determine how postzygotic isolation evolves as a consequence of adaptation, the relative importance of extrinsic and intrinsic barriers, and how postzygotic isolation may act alone or in concert with prezygotic isolation to cause ecological speciation.

## Figures and Tables

**Table 1a:** (a) Latency to mate

	Outlier included			Outlier removed		
Source	*df *	* F*	*P*	*df *	* F*	*P*

Male population	1,28	0.32	0.58	1,27	0.34	0.56
Female population	1,28	0.52	0.48	**1,27**	**9.31**	**0.0051**
Male ∗ Female population	1,28	1.17	0.29	1,27	0.18	0.68

**Table 1b:** (b) Total number of eggs produced

Source	*df *	* F*	*P*
Male population	1,28	0.08	0.78
Female population	1,28	<0.01	0.96
Male ∗ Female population	1,28	2.57	0.12

**Table 2a:** (a) Proportion of eggs hatched

Source	*df *	*χ* ^2^	* P*
*Cross*	**2**	**18.83**	**<0.0001**
*Water Chemistry*	**4**	**106.48**	**<0.0001**
Cross ∗ Water Chemistry	8	8.23	0.4116

**Table 2b:** (b) Proportion of fry survive

Source	*df*	*χ* ^2^	* P*
Cross	2	0.64	0.7257
*Water Chemistry*	**4**	**59.73**	**<0.0001**
*Cross* ∗ *Water Chemistry *	**8**	**24.77**	**0.0017**

**Table 2c:** (c) Total survival

Source	*df *	*χ* ^2^	* P*
Cross	2	1.83	0.4013
*Water Chemistry*	**4**	**77.47**	**<0.0001**
*Cross* ∗ *Water Chemistry *	**8**	**19.90**	**0.0107**

**Table 3a:** (a) Proportion of eggs hatched

Source	*df *	*χ* ^2^	* P*
*MB*	**1**	**51.91**	**<0.0001**
*Water Chemistry*	**1**	**35.45**	**<0.0001**
*MB* ∗ *Water Chemistry *	**1**	**29.80**	**<0.0001**
*Cross*	**2**	**4.27**	**0.014**
*MB* ∗ *Cross *	**2**	**5.39**	**0.0045**
Water Chemistry ∗ Cross	2	2.02	0.1325
MB ∗ Water Chemistry ∗ Cross	2	0.13	0.8821

**Table 3b:** (b) Proportion of fry survive

Source	*df *	*χ* ^2^	* P*
MB	1	0	0.9574
Water Chemistry	1	1.41	0.2357
MB ∗ Water Chemistry	1	0.12	0.7278
Cross	2	0.96	0.3828
MB ∗ Cross	2	0.07	0.9285
*Water Chemistry* ∗ *Cross *	**2**	**7.06**	**0.0009**
*MB* ∗ *Water Chemistry* ∗ *Cross *	**2**	**4.58**	**0.0103**

**Table 3c:** (c) Total survival

Source	*df *	*χ* ^2^	* P*
*MB*	**1**	**56.55**	**<0.0001**
*Water Chemistry*	**1**	**22.27**	**<0.0001**
*MB* ∗ *Water Chemistry *	**1**	**12.04**	**0.0005**
Cross	2	2.74	0.0644
MB ∗ Cross	2	2.85	0.0578
*Water Chemistry* ∗ *Cross *	**2**	**4.31**	**0.0135**
MB ∗ Water Chemistry ∗ Cross	2	2.67	0.0692

**Table 4a:** (a) Proportion of eggs hatched

Source	*df *	*χ* ^2^	* P*
*Cross*	**3**	**27.94**	**<0.0001**
*Water Chemistry*	**1**	**7.26**	**0.0070**
Cross ∗ Water Chemistry	3	5.38	0.1452

**Table 4b:** (b) Proportion of fry survive

Source	*df *	*χ* ^2^	* P*
Cross	3	4.02	0.2592
Water Chemistry	1	0.62	0.4302
Cross ∗ Water Chemistry	3	6.47	0.0908

**Table 4c:** (c) Total survival

Source	*df *	*χ* ^2^	* P*
*Cross*	** 3**	**9.48**	**<0.0001**
Water Chemistry	1	1.53	0.2162
*Cross* ∗ *Water Chemistry *	**3**	**3.25**	**0.0209**
